# Innovative lipid delivery systems in ruminant diets: the role of spray drying

**DOI:** 10.3389/fvets.2025.1619263

**Published:** 2025-11-14

**Authors:** Thâmilla Thalline Batista de Oliveira, Thalia Catherine Sacramento Ferreira, Pedro Paulo Lordelo Guimaraes Tavares, Paulo Romano Cruz Correia, Cláudio Vaz Di Mambro Ribeiro, Carolina Oliveira de Souza

**Affiliations:** 1 Postgraduate Program in Food Science, Faculty of Pharmacy, Federal University of Bahia, Salvador, Brazil; 2 School of Veterinary Medicine and Animal Science, Federal University of Bahia, Salvador, Brazil

**Keywords:** animal supplementation, ruminants, lipids, tannins, microencapsulation

## Abstract

Ruminant nutrition faces multifactorial challenges, including genetic limitations, inadequate management practices, and biochemical and physiological constraints within the ruminal environment, which compromise nutrient utilization, animal productivity, and the environmental sustainability of production systems. One of the main bottlenecks is microbial biohydrogenation of lipids, which reduces the energy efficiency of diets and the bioavailability of essential fatty acids in ruminants. Spray-drying-based microencapsulation technology has been proposed as a strategy to protect lipids and bioactive compounds. However, comprehensive mechanistic reviews integrating scientific evidence, technological development, and zootechnical applicability are still scarce. This systematic review aims to map the landscape of scientific literature and technological records regarding the use of microencapsulated lipids and tannins in ruminant feeding, with an emphasis on spray-drying as the primary microencapsulation technique. The methodology involved bibliometric and technological analyses using The Lens database, covering publications and patents from 2015 to 2025. A total of 1,190 patents and 163 scientific articles on fatty acid microencapsulation were identified, highlighting efforts to improve thermal and oxidative stability, control ruminal release, and increase the dietary energy efficiency. Regarding tannins, 161 patents and 29 studies emphasized their role as co-microencapsulants with antioxidant, antimicrobial, and fermentation-modulating properties. A scarcity of applied studies in tropical areas was observed, along with a geographic mismatch between areas of high technological output, such as Europe, and major ruminant-producing countries, such as Brazil and India, highlighting the need for regionally adapted innovation. The mapped landscape underscores the strategic and multidimensional potential of microencapsulation technologies to mitigate nutritional losses, reduce enteric emissions, and promote efficient livestock systems. This review provides a critical and updated analysis of the trends, barriers, and opportunities, offering insights into the pathways for sustainable innovation in ruminant nutrition. These findings underscore the importance of aligning scientific advances with practical solutions in animal production.

## 1 Introduction

Ruminants have developed specialized anatomical and genetic characteristics through evolutionary processes to adapt to various aspects of survival, including nutritional conditions that are critical for energy acquisition and growth. The ruminant microbiota harbors microorganisms that facilitate essential reactions for nutrient extraction from the feed (1). However, the interaction between these microorganisms and lipids, a primary energy source for ruminants, can significantly influence animal metabolism and productive performance, as fatty acid biohydrogenation in the rumen alters the availability and utilization of these compounds (2).

In addition to genetics, management practices substantially influence ruminant nutrition. Typically, ruminant diets consist primarily of forage with low lipid content, which fails to provide sufficient energy for optimal metabolism (3). Consequently, nutritional management strategies must balance dietary supplementation with the maintenance of rumen health, as metabolic disorders often arise from intensive feeding practices rather than natural rumen processes. Proper nutrition not only improves digestibility but also reduces greenhouse gas emissions, particularly methane, released by ruminants (4). These challenges are particularly pronounced in tropical production systems, where forage quality, environmental stressors, and limited access to advanced feed technologies exacerbate nutritional inefficiencies (5). In such settings, feed innovations must strike a balance between environmental constraints, economic feasibility, and regional adaptability to ensure their successful implementation.

Ruminant nutrition profoundly affects animal health, the quality of derived products, and consequently, consumer food safety. As one of agribusiness’s most significant activities, it exerts socioeconomic impacts on farming systems (6). Given this relevance, research aimed at improving feeding conditions for this animal group has gained prominence, with scientific efforts seeking to harmonize the synergy between genetics, management, and nutrition to ensure animal health and, consequently, improve product quality.

Among the strategies and innovations studied, the incorporation of feed additives in ruminant diets has the potential to enhance rumen metabolic efficiency, with positive impacts on quality, health, and management conditions. Exploring technologies that promote these improvements in animal nutritional status is therefore crucial (7). However, the stability, bioavailability, and targeted release of these additives remain technical bottlenecks, especially in the complex and degradative ruminal environment. This highlights the need for encapsulation systems capable of protecting and modulating the release of active compounds.

Microencapsulation technology has emerged as a promising tool that enables substances to be enclosed within capsules of macro (>100 μm), micro (1–100 μm), or nano (1–100 nm) dimensions, thereby protecting compounds from external physical, chemical, or biological challenges (8). This protection is provided by wall materials, polymers, proteins, or polysaccharides that form a protective matrix around the core compound, that control the release of compounds in the digestive tract, responding to changes in pH or temperature, and optimizing effectiveness (9). Among microencapsulation methods, the spray-drying technique, which converts emulsions into powder through spraying and hot drying, is notable for its industrial viability, high yield, and cost-effectiveness (9). Its widespread application in the food industry has been explored as a supplement to feeds with nutrients lacking in conventional diets, such as lipids. When microencapsulated, these compounds are protected against microbial degradation in the rumen, promoting more efficient digestibility and enhanced nutritional gain (10).

In ruminant nutrition, microencapsulation can supplement relevant nutrients in conventional feed, enabling complete nutrition (11). For instance, microencapsulation of lipids protects fatty acids from degradation by the rumen microbiota, promoting more efficient feed digestibility and improved nutrient utilization (Besharati, 2024). The protective barrier, referred to as the wall material, must be selected based on its potential to effectively coat the compound of interest, in this case, lipids. Gum arabic and maltodextrin are commonly used for this purpose (12). Recently, tannins have been investigated for their ability to protect dietary proteins from rumen degradation, although further studies are needed to assess their stability, sensory properties, and bioavailability (13). The potential benefits of tannins as co-microencapsulating agents with bioactive properties, such as antioxidant, antimicrobial, and fermentation-modulating effects, adds a novel dimension to ruminant feed formulations. This synergistic combination of energy-dense lipids and functional polyphenols, such as tannins, may represent an emerging frontier in precision feeding systems (14). However, it remains insufficiently characterized in the current literature and lacks a systematized mapping of its development trajectory.

Despite these advances, a comprehensive mechanistic understanding of the interactions between microencapsulated lipids and bioactive compounds, such as tannins, in the rumen environment remains lacking. Although progress in microencapsulation has been reported, there is limited evidence on how this technology is being developed and contextualized for tropical livestock systems, where production realities and nutritional constraints differ significantly from those in temperate regions. This disparity limits the scalability and relevance of current innovations, underscoring the importance of aligning technological development with the practical demands of local production systems.

Therefore, this study aimed to conduct a systematic bibliometric and technological review to map the evolution, distribution, and scientific and technological advances related to the use of microencapsulated lipids and tannins in ruminant nutrition. It identifies temporal trends, geographical distributions and knowledge gaps. These insights reveal innovation trajectories and their alignment with sustainable and high-performance livestock systems. Furthermore, this review offers an unprecedented integration of scientific literature and patent data, providing a comprehensive perspective on how knowledge is generated, disseminated, and translated into applicable solutions.

## 2 Review methodology

This research employed a qualitative–quantitative approach, based on quantifying, analyzing, and interpreting data obtained from a survey of scientific publications and patents on The Lens database platform, an open knowledge resource that integrates scholarly and patent data to support transparent innovation and research analyses (15). With an applied character and exploratory objective, the bibliometric research utilized a combination of English keywords related to the topic and Boolean operators to direct the searches.

Initially, to assess the general landscape, a search for “ruminants” and “nutrition” was conducted without period restrictions or filters. This preliminary exploration revealed that the earliest patent and publication records on ruminant nutrition dated back to the 1950s, marking the first significant increase in research activity in this field. Subsequently, other combinations were explored.

**Figure 1:**
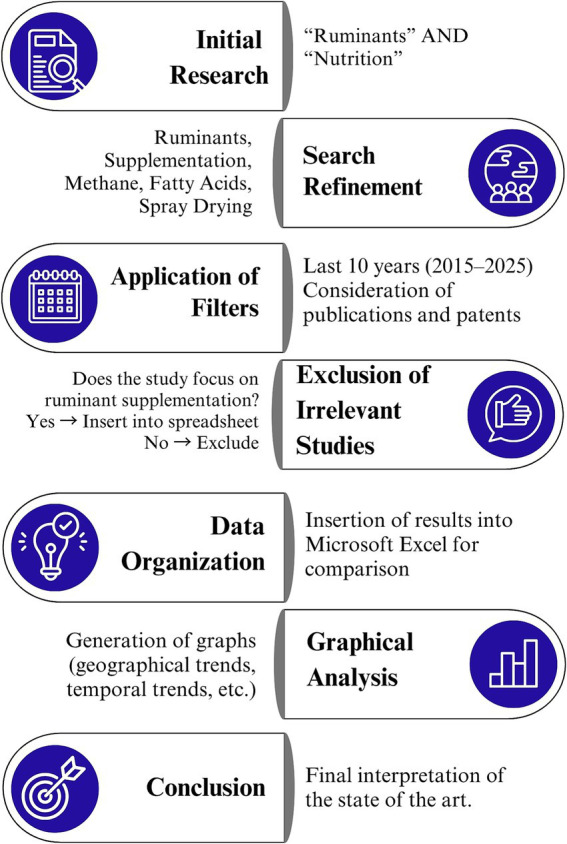
Systematic representation of the applied methodology.

The research, therefore, consisted of searches for topics related to nutrition, ruminant supplementation, and methane mitigation. The scope was subsequently refined by including specific terms such as microencapsulation by spray drying, lipids, and the use of tannins, applied to both patents and scientific articles. From this delimitation, only studies aligned with the objective of ruminant feed supplementation were considered. At the same time, those addressing other methods of animal nutrition or health, as well as alternative approaches to methane mitigation (e.g., vaccines and technologies aimed at feed administration or distribution), were excluded. The methodological steps are summarized in Figure 1.

Specific criteria were defined for database platform searches, including “Field” parameters for publications and “Grouping” parameters for patents. The “Field” criterion delimited keyword search locations (title, abstract, claims, keywords, or full text), while the “Grouping” criterion filtered patent documents originating from the same initial document. Additionally, the publication period was restricted to the last decade (2015–2025) to assess academic and technological trends related to the study subject.

During the survey process, studies identified as most relevant and aligned with the research objective were systematically organized in Excel spreadsheets.

This organization aimed not only to facilitate comparative analysis and inference of data obtained at each search stage but also to consolidate an overview of the state of the art on the subject. Additionally, platform-generated graphs contributed to more in-depth analyses, providing visual perspectives on relevant aspects, such as primary geographical publication regions, the temporal evolution of deposit numbers, the most influential journals and authors, involved institutions, and the legal status of identified patents. This approach complemented the bibliographic and patent surveys, promoting a comprehensive and systematic understanding of technological and scientific trends associated with the topic under study.

## 3 Results and discussion

### 3.1 Overview of ruminant nutrition research

The pivotal role of ruminant nutrition in livestock systems is evident not only in its impact on productivity and product quality but also in its growing presence in the scientific and technological landscape of livestock production. The number of publications and patents reflects a steady global interest, reinforcing the fact that nutritional strategies are central to enhancing animal performance and sustainaility. This increasing volume of intellectual production may indicate a demand for more refined and targeted feeding practices, particularly considering the constraints imposed by climate change and competition for feed resources.

Searching for “ruminants” and “nutrition” in the database yielded 25,220 publications and 14,786 patents, dating back to 1956, such as patent document GB 866924 A, which describes a method of supplementing ruminants by administering pellets composed of biologically active substances for release into the rumen-reticulum (16). These developments laid the groundwork for technological strategies currently applied in lipid supplementation, particularly those aimed at controlling the release of bioactive compounds in the rumen and enhancing post-ruminal absorption, such as microencapsulation.

The surge in research in the 1950s aligned with a global shift toward livestock intensification. Scientific attention has focused on understanding ruminal microbial metabolism, particularly cellulose digestion and microbial protein synthesis, as these processes are pivotal for improving feed conversion ratios, which is a key parameter of animal production efficiency (17). Simultaneously, innovations such as non-protein nitrogen supplementation and hormonal growth promoters have reflected an increasing reliance on nutritional and pharmacological strategies to enhance performance metrics, such as weight gain and milk yield (18). The expanding demand for meat and milk has also necessitated more efficient feeding strategies, which has led to increased investment in digestive physiology and the formulation of balanced diets (19). These developments laid the conceptual and technological groundwork for modern approaches that aim to optimize rumen fermentation and nutrient bioavailability, which underpins the rationale for technologies such as lipid microencapsulation designed to protect compounds from ruminal microbial degradation, thereby enhancing nutrient availability beyond the rumen.

By restricting the search to the last decade and including specific terms, the results shown in Table 1 were obtained. The analysis revealed a significant volume of studies on ruminant nutrition, emphasizing supplementation, fatty acids, and lipid microencapsulation, which reflect the primary technological and scientific trends in the field.

**Table 1:** Results of patents and publications according to research conducted on the lens.

Search	Keywords	Patents	Scientific articles
1	Ruminant AND Nutrition AND Supplementation	2,321	4,565
2	Ruminant AND Nutrition AND Supplementation AND Methane	427	936
3	Ruminant AND Nutrition AND (fatty acids)	1932	3,574
4	Ruminant AND Nutrition AND (fatty acids) AND (spray drying)	1,190	163
5	Ruminant AND Nutrition AND (fatty acids) AND (spray drying) AND Tannin	161	29
6	Microencap* AND Ruminant AND Lipids	239	36
7	Microencap* AND Ruminant AND Lipids AND Tannin	9	10

Table 1 highlights the prominence of nutritional supplementation in the research. This focus reflects the persistent challenge of formulating efficient diets under diverse environmental conditions (20). Notably, the parallel emphasis on methane mitigation illustrates how nutrition is viewed from an environmental perspective. Methane, a product of enteric fermentation, represents both environmental liability and energetic inefficiency in ruminant production systems (21). The integration of additives, such as tannins and essential oils, suggests a growing interest in functional ingredients with dual benefits: enhancing productivity while modulating fermentation to reduce greenhouse gas emissions (14, 22). Tannins, in particular, can form complexes with proteins and lipids, thereby reducing their degradation by ruminal microbes and shifting fermentation toward less methanogenic pathways (23–25). However, the synergistic use of lipids and tannins remains poorly explored in experimental studies, especially under tropical production conditions, where dietary composition and environmental stressors differ significantly from those in temperate regions. This gap highlights the need for targeted research that addresses region-specific applications and the long-term effects of such combinations on animal performance and environmental outcomes (22, 23, 26).

Fatty acids are another important topic, particularly in relation to the spray-drying process. This method is widely used to stabilize lipids and increase their bioavailability in animal diets (10). Although still relatively unexplored, the combination of this technique with tannins suggests an emerging research niche that could offer innovative solutions for ruminant nutrition.

Lipid microencapsulation has proven to be a promising strategy that allows greater control over nutrient release into the rumen, reduces losses, and improves absorption efficiency. The combined use of microencapsulation and tannins, while still in its early stages, shows potential for new developments, possibly aimed at protecting bioactive compounds in the animal digestive tract.

The dual presence of academic and technological outputs indicates that ruminant nutrition is not merely a theoretical concern but a strategic priority within innovation pipelines. This convergence highlights the increasing alignment between the scientific community and livestock industry in developing strategies to improve feed efficiency, animal health, and environmental sustainability. However, despite the increasing volume of publications and patent filings, the translation of findings into scalable and cost-effective technologies, such as spray-dried microencapsulated lipids, remains constrained by challenges related to process optimization, compound stability, and variability in field conditions (10, 27). This gap reinforces the need for translational research that not only refines encapsulation methodologies but also validates them under commercial-scale conditions. Accordingly, the bibliometric-technological mapping presented herein aims to bridge this divide by identifying key research trends, patent activities, and underexplored synergies, ultimately contributing to the development of robust and context-sensitive solutions for ruminant systems.

These findings highlight the growing integration of scientific and technological approaches in ruminant nutrition, particularly fatty acid microencapsulation. Nonetheless, the limited number of integrative studies that consolidate and critically assess this technological trajectory reinforces the relevance of bibliometric mapping as a strategic tool to guide future innovations and practical applications in ruminant nutrition.

### 3.2 Environmental challenges and methane mitigation

Methane, a primary greenhouse gas (GHG), is a short-lived gas with a significantly higher global warming potential than carbon dioxide, especially over shorter timeframes (28). According to the Intergovernmental Panel on Climate Change, this gas accounts for approximately 14–16% of the total global GHG emissions (29, 30). The livestock industry contributes substantially to these emissions, releasing approximately 7.1 gigatonnes of carbon dioxide equivalent annually, corresponding to approximately 14.5% of all human GHG emissions globally (31). Given the significance of these emissions, methane mitigation has become a priority in sustainability and efficiency strategies for livestock production, highlighting the importance of an integrated approach that involves animal nutrition, production system modifications, and utilization of innovative technologies.

Methane is a product of rumen fermentation. In addition to contributing to global warming, its evacuation indicates energy losses during digestion. To reduce methane production and its consequences, animal diets can be modified by altering carbohydrates, incorporating lipid supplementation, or adding feed additives (32). Strategies are therefore being continuously developed and tested to achieve this goal.

According to Search 2 (Table 1), 427 patents and 936 publications were identified that expressed environmental concerns linked to ruminant nutrition. For example, patent EP 4000411 B1 describes a method and composition for reducing ruminant methane production by incorporating a silage-based nutritional supplement into the animal diet. This composition comprises beet pulp, wheat gluten feed, maize silage, spent grain, and grass clippings (33). The patent indicates the potential to effectively reduce methane emissions in ruminants without compromising digestibility or energy efficiency, directly addressing the need for sustainable strategies in ruminant feeding to mitigate greenhouse gas emissions in the long term. This approach aligns with current trends that seek to increase productivity and ensure environmentally responsible farming practices (34, 35).

In contrast, US document 2022/0192229 A1 describes a methane mitigation method involving the oral administration of an enzyme inhibitor, 3-nitrooxypropanol, from birth to 6 months of age, resulting in at least a 10% reduction in methane after 1 year of age (84). This example contrasts with feed-based strategies relying on natural ingredients, as it represents a targeted chemical approach. In this context, microencapsulation is considered a benchmark strategy for nutrient protection and controlled release, against which alternative nutritional and technological approaches can be compared.

In research conducted by Akinbode et al. (36) supplemented goat diets with guava leaves and assessed *in vitro* methane production, degradability, and protozoal population. The study found that supplementation improved the crude protein content and resulted in a 59% reduction in methane and protozoal counts when the diet contained 30% guava leaves. This finding illustrates how plant-derived additives can alter fermentation dynamics by modifying microbial populations and favoring propionate production over acetate, thereby decreasing the availability of hydrogen for methanogenesis (14, 22).

Although diverse in formulation, these approaches share a common principle: alteration of ruminal fermentation pathways through nutritional interventions or targeted inhibition of methanogenic archaea. However, despite methane mitigation, feed additives such as fatty acids or alternative forage sources fail to provide sufficient energy or adequately regulate the rumen microbiota (37).This underscores the limitations of isolated interventions and reinforces the need for integrative strategies that combine mitigation efficacy with nutritional adequacy.

Beyond reducing fermentation thermal stress, fatty acids enhance vitamin absorption, improve palatability, and promote overall digestion, while increasing energy density and modulating rumen fermentation through the antimicrobial effects of unsaturated fatty acids (37). Consequently, lipid supplementation stands out as a source of these fatty acids, which are essential for regulating energy metabolism and animal cognitive, behavioral, and physiological development (38).

In addition, long-chain unsaturated fatty acids can inhibit the growth of methanogenic archaea and protozoa, resulting in decreased methane production; however, their effects depend on the dose, lipid source, and interaction with diet composition (39).

Studies comparing lipid sources (canola vs. coconut oil) have demonstrated differential methane suppression, reinforcing the importance of selecting lipid profiles that maximize fermentation modulation without impairing fiber digestibility (20, 40). However, the lack of consistency *in vivo* highlights a methodological gap and the need for standardized evaluation frameworks.

Furthermore, few studies have evaluated the long-term impacts of these strategies under commercial tropical conditions, where heat stress, forage type, and breed variability can affect both methane output and dairy cow performance. This reinforces the urgency of regionally adapted trials that integrate climate, productivity, and economic feasibility into the methane mitigation equation.

Given these advances, mitigating methane emissions presents a multifaceted challenge involving technological innovations, nutritional strategies, and critical analysis of available alternatives. Combining different approaches can lead to more sustainable and efficient solutions, benefiting both animal production and the environment.

Future research should prioritize combinatory trials that evaluate the synergistic effects of lipids, tannins, and enzyme inhibitors under practical conditions, supported by life-cycle assessments and cost–benefit analyses to guide adoption by farmers and industry.

### 3.3 Fatty acids and microencapsulation in ruminant nutrition

Fatty acid supplementation provides direct benefits to ruminant health while significantly enhancing the entire production chain, from breeding to final consumption. These essential fatty acids must be obtained through diet, as humans and animals cannot naturally synthesize them (10). Therefore, the appropriate use of these compounds in animal feed contributes to both productivity and sustainability of the production system, positively impacting animal product quality and the environment.

The applicability of fatty acids in ruminant feed is evident from the Search 3 results (Table 1), with 1,932 patents and 3,574 publications during the research period, demonstrating their significance through their biological functions. However, a significant challenge in fatty acid supplementation is the deterioration caused by lipid oxidation, a spontaneous process that leads to loss of functional activity and sensory alterations (41). Additionally, due to rumen microbial load, fatty acids undergo structural changes via hydrolysis and biohydrogenation processes (42).

These biochemical transformations result in the saturation of unsaturated fatty acids, potentially reducing the nutritional value and desired health effects of final animal products, such as milk and meat. Biohydrogenation, for instance, transforms beneficial polyunsaturated fatty acids (PUFAs), such as linoleic acid, into less bioactive saturated forms, thereby minimizing their functional benefits (43, 44). Thus, stabilizing these molecules before they reach the small intestine is a technological objective.

These challenges have motivated the search for methods that promote stability and site-specific (post-rumen) release of fatty acids, particularly through microencapsulation technology, which is widely applied to enhance the stability of compounds (45). Consequently, lipid matrix microencapsulation has become a reference for delivering nutrients to the ruminant small intestine, as evidenced by 1,990 patents and 163 publications in Search 4 and 239 patents and 36 publications in Search 6, respectively (Table 1).

Microencapsulation provides a protective barrier for bioactive lipids, shielding them from rumen microbes and minimizing unwanted transformations. The selection of wall materials, such as polysaccharides, proteins, and polyphenols, significantly influences microencapsulation efficiency and release kinetics (10). For example, using whey protein isolate and gum arabic in complex coacervates, tuna oil rich in omega-3 PUFAs was microencapsulated, resulting in solid microparticles with higher oxidative stability, greater encapsulation efficiency, and reduced surface oil, defined as the non-encapsulated oil remaining on the particle surface, which is critical because it accelerates lipid oxidation and decreases storage stability, compared to freeze-dried systems (46). This technique also enables targeted release in the intestine, enhancing fatty acid absorption and bioavailability.

Another relevant aspect is that microencapsulation extends beyond fatty acids. Compounds such as fat-soluble vitamins, probiotics, antioxidants, and veterinary medicines can benefit from this approach (47). Microencapsulation protects these compounds from the action of digestive enzymes and, in some cases, from light or high-temperature conditions, offering more efficient and controlled supplementation with positive effects on ruminant health and productivity (27).

Furthermore, microencapsulated nutraceuticals may interact synergistically, as shown in combinations of fatty acids and polyphenols, such as tannins, which not only reduce ruminal degradation of nutrients, but may also exhibit anti-inflammatory or methane-mitigation effects (14, 23). This suggests a promising research direction: exploring microencapsulated systems tailored to specific physiological targets.

Among microencapsulation techniques, spray drying has attracted significant interest as the most suitable method for drying emulsions containing lipid and water sources, as demonstrated by Mohammadi et al. (48), who compiled articles on the micro- and nanoencapsulation of conjugated linoleic acid and ruminant trans fatty acids to reduce the risk factors associated with metabolic syndrome. Similarly, Berraquero-García et al. (49) identified this technique as the most commonly used, given its ability to trap bioactive and thermosensitive compounds at moderate temperatures without degradation. Additionally, it delivers dry microparticles cost-effectively, with scalability potential, and the possibility of double-layer protection.

Spray drying offers key operational advantages for lipid microencapsulation, including the ability to enable short residence times, control droplet size, and moderate drying temperatures, which help preserve thermosensitive compounds, such as polyunsaturated fatty acids (PUFAs) (50). These factors contribute to high process efficiency and scalability, making spray drying the preferred method in industrial applications. However, limitations still exist regarding the oxidative stability and microencapsulation yield, especially for highly unsaturated oils. To address these challenges, formulation strategies involving emulsifiers and antioxidants have been shown to enhance emulsion stability and protect the encapsulated core from degradation during and after drying (51, 52).

In parallel with improvements in conventional spray drying, alternative drying technologies have been investigated to overcome their limitations in terms of structural protection and oxidative control. Among these, spray freeze-drying (SFD) has emerged as a promising strategy because of its ability to produce highly porous and stable microparticles. Although technically advantageous, SFD is currently limited to experimental or high-value applications. This restriction is primarily due to elevated operational costs and low processing yields, which necessitate substantial investments in specialized equipment and cryogenic infrastructure. These economic and logistical constraints hinder its large-scale industrial use, despite its considerable potential for enhancing the stability of microencapsulated lipids and other bioactives (53).

### 3.4 Innovation with tannins in microencapsulation

The choice of microencapsulation technique is crucial, as it influences the yield, microparticle morphology, and production costs (54). Similarly, the process conditions and type of wall material affect the bioavailability of active compounds and system efficiency (55). Consequently, studies have focused on developing microencapsulated functional lipids based on wall materials that not only protect active compounds but also enrich food products incorporating microparticles, thereby contributing additional benefits such as improved thermal stability and humidity protection, which potentially extend the final product’s shelf life (56).

In this context, natural polyphenolic compounds, such as tannins, have emerged as promising wall components owing to their dual role as structural stabilizers and functional additives. Building on their well-documented effects in ruminant nutrition, tannins have long been investigated for their ability to modulate ruminal fermentation dynamics and improve nitrogen utilization efficiency. Their inclusion in conventional diets has been shown to decrease proteolytic activity and reduce excessive protein degradation through the formation of tannin–protein complexes, thereby improving feed conversion and nitrogen retention (13, 14, 57).

Evidence from encapsulation studies supports the hypothesis that tannins enhance encapsulation efficiency, matrix stability, and the bioavailability of active compounds. Inô et al. (24) demonstrated that incorporating natural tannins into lipid matrices used for rumen-protected lysine increased microencapsulation efficiency and retention of the active compound compared with formulations without tannins. Similarly, Adejoro et al. (58) reported that tannin-enriched lipid microparticles exhibited higher encapsulation efficiency and slower release rates under varying pH conditions, indicating improved structural stability. These effects are attributed to the ability of tannins to form hydrogen bonds and hydrophobic interactions with proteins and lipids, resulting in stable matrices that resist ruminal degradation and promote the intestinal delivery of bioactive compounds (14, 32, 57). Furthermore, *in vitro* and *in vivo* studies have shown that lipid–tannin complexes reduce lipid hydrogenation, enhance fatty acid absorption, and preserve the functional integrity of PUFAs, demonstrating their potential as formulation adjuvants for ruminant nutrition (58).

Beyond modulating rumen metabolic processes, tannins exhibit antimicrobial and antioxidant properties that contribute to nutrient preservation and redox balance in ruminant systems (24). Their antioxidant activity occurs mainly within the digestive environment, where they scavenge reactive oxygen species and inhibit lipid and protein oxidation, thereby preventing oxidative chain reactions. These local effects indirectly support systemic antioxidant defense and animal health by reducing oxidative stress and inflammation (59–61). Furthermore, the antimicrobial properties of tannins may reduce undesirable ruminal fermentation pathways, such as ammonia production and methanogenesis, in line with sustainable livestock strategies ((59); Adejoro, Hassen & Thantsha, 2018).

These functionalities position tannins as promising agents for enhancing ruminant lipid supplementation; however, their application in microencapsulation remains relatively unexplored. This is evidenced by the survey results, which identified 161 patent applications and 29 publications in Search 5, as well as only nine patents and 10 publications in Search 7 (Table 1). These figures underscore the early developmental stage of this technological approach and reveal critical research gaps in formulation standardization, mechanistic understanding, and large-scale validation of the technology.

Patent filings corroborate the growing interest in biotechnological strategies for mitigating methane emissions and improving ruminal efficiency in livestock. Fitch et al. (62) describe compositions and methods for reducing methane emissions in ruminant populations, employing formulations that combine bioactive compounds and delivery mechanisms to modulate the rumen microbiota and suppress methanogenic pathways (WO 2024/015366 A1). Similarly, South et al. (63) disclose a related system involving biologically active additives and protective carriers designed to maintain compound stability and efficacy during ruminal passage (US 2024/0189408 A1). Both patents emphasize performance improvement and environmental benefits, aligning with the broader movement toward sustainable animal nutrition. However, these disclosures remain primarily conceptual, presenting mechanistic hypotheses without quantitative *in vivo* validation. This reinforces our conclusion that the field is still at an early developmental stage, characterized by promising formulations but insufficient standardized evidence regarding efficacy, stability, and large-scale applicability.

To address these limitations and unlock the full potential of tannin-based systems, future studies should prioritize the development of advanced encapsulation strategies, such as multilayer capsules and nanoemulsions, by combining tannins with conventional polymers and systematically evaluating the effects of tannin type and molecular structure (condensed vs. hydrolyzable) on release kinetics and lipid protection. Furthermore, conducting both *in vitro* and in vivo studies is valuable for understanding how tannins influence fatty acid digestibility, contribute to methane mitigation, and affect the performance of animals. Such evaluations play a crucial role in supporting the application of these aditives to enhance the stability and bioavailability of functional compounds in ruminant diets.

### 3.5 Geographical analysis and research trends

Lipid microencapsulation with tannins has been associated with significant benefits, including methane mitigation and improved digestibility and palatability of compounds in ruminants. In this context, Ibrahim and Hassen (64) investigated the influence of non-microencapsulated and microencapsulated Mimosa tannins in sunflower oil on growth performance, digestibility, methane emissions, and rumen fermentation in South African Merino sheep. They also assessed how microencapsulation technology contributes to neutralizing astringency and ensuring controlled tannin solubility in the ruminant digestive tract. Through physicochemical and in vivo analyses, the authors demonstrated that tannin microencapsulated with sunflower oil, a lipid source, promoted an average reduction of approximately 20% in enteric methane emissions when supplemented at 20 g/kg of feed dry matter, without affecting digestibility. Additionally, microencapsulation masked the characteristic astringent flavor of tannins, enhancing their acceptability to consumers.

**Figure 2:**
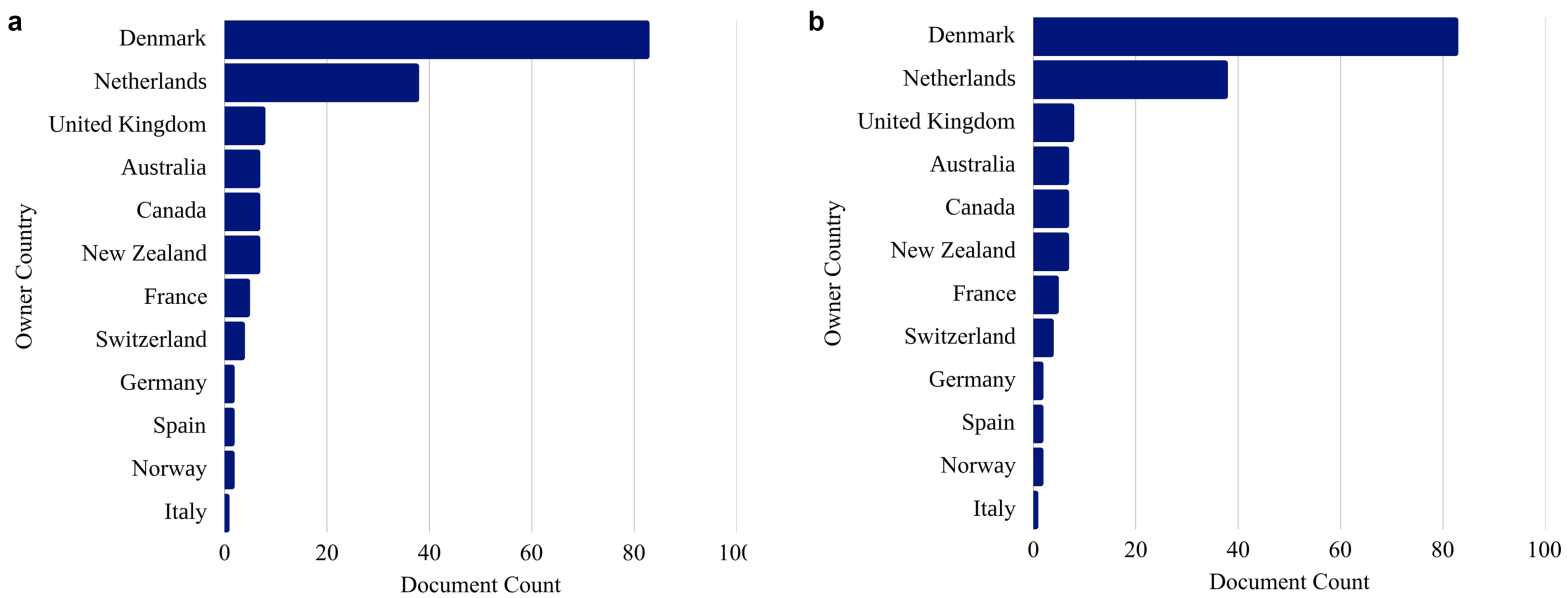
**(a)** Graph of patent document filings by country between 2015 and 2025 (based on Search 5). Source: The Lens, 2025. **(b)** Graph of scientific publication filings by country between 2015 and 2025 (based on Search 5). Source: The Lens, 2025.

The proposal to supplement ruminant diets with tannin-containing microencapsulated lipids addresses the specific nutritional requirements of these animals while promoting socio-environmental benefits by reducing environmental impact. Most initiatives for this proposal originate from European countries, including Denmark, the Netherlands, the United Kingdom, and Germany, as illustrated in Figures 2A,B.

While European countries lead in scientific publications and patent registrations, the global landscape of ruminant production presents a striking contrast. Brazil, India, the United States, and China are the primary countries involved in ruminant farming (65). Brazil alone accounted for over 238 million head of cattle in 2023 and ranks as the world’s largest beef exporter, underscoring its strategic importance in livestock production (66). This geographical dissociation between research output and production volume reveals a critical gap, particularly because regional variables, such as genetic diversity, feeding strategies, and environmental conditions, directly influence the effectiveness of technologies such as lipid microencapsulation (23, 24, 67). Bibliometric analyses have confirmed that although countries such as China, Brazil, and India are increasing their scientific output on ruminant methane mitigation and feed efficiency, they still exhibit higher emission intensities per unit of product than industrialized nations, reinforcing the need for localized technological validation (68, 69). Moreover, the integration of microencapsulation technologies into regional production systems remains limited because of the scarcity of field-scale trials and insufficient cost–benefit assessments under tropical or semi-arid conditions (8, 64). Part of this gap also reflects infrastructure and cost-related constraints: in many developing regions, access to pilot-scale spray-drying and emulsification equipment, as well as analytical facilities such, essential for assessing encapsulation efficiency, oxidative stability, and compound release, is restricted to a few academic centers. This lack of infrastructure limits process optimization and validation under realistic production conditions. Additionally, the high operational costs of these systems, coupled with limited industry–academia integration and weak technology transfer mechanisms, further delay the translation of laboratory research into scalable and economically viable applications. Therefore, aligning innovation efforts with local production contexts is essential to ensure that scientific advances not only enhance animal performance and reduce emissions but also generate a scalable and sustainable impact in major livestock-producing regions.

**Figure 3:**
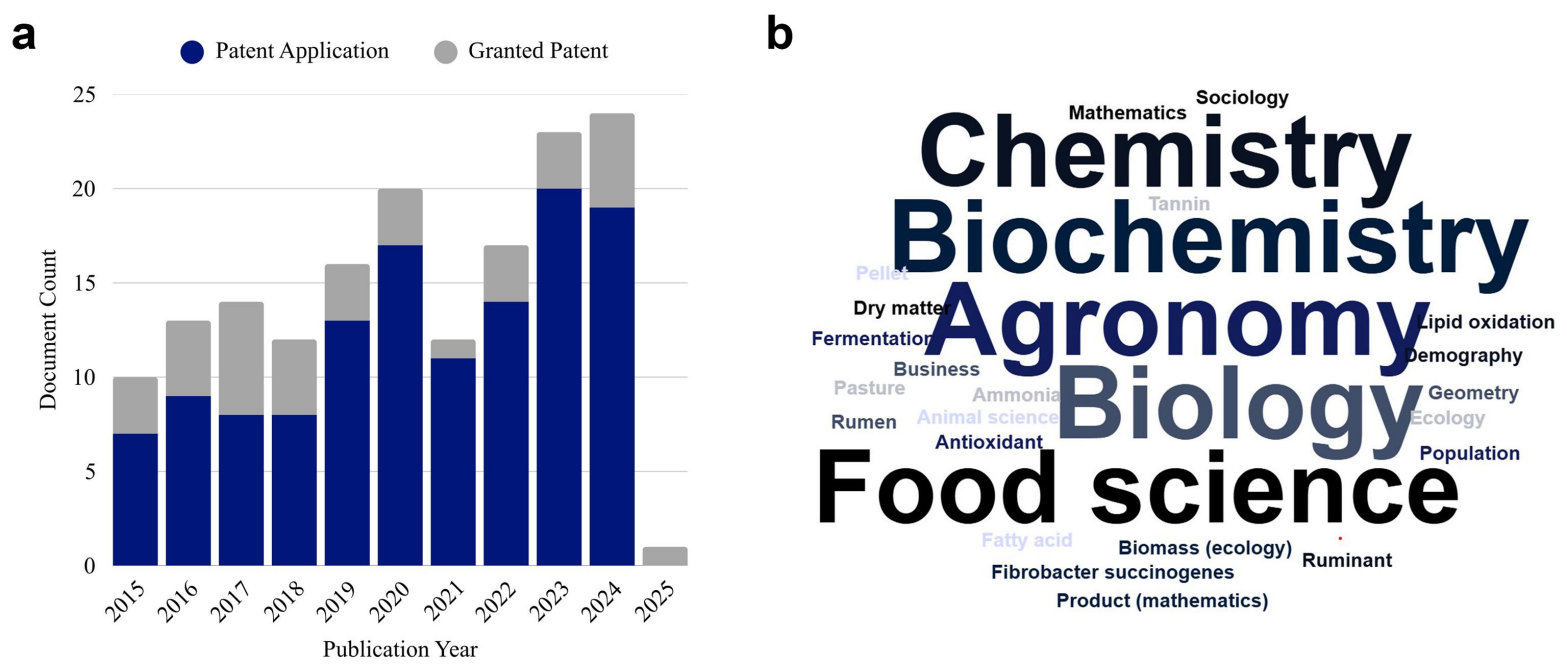
**(a)** Patent documents related to lipid microencapsulation with tannins filed between 2015 and 2025 (based on Search 5). Source: The Lens, 2025. **(b)** Keywords identified in scientific publications related to lipid microencapsulation with tannins between 2015 and 2025 (based on Search 5). Source: The Lens, 2025.

Another perspective for evaluation is the disparity between patent numbers and academic publications, as shown in Figures 3A,B. The analysis of Figure 3A reveals a significant trend in the number of granted patents and patent applications related to lipid microencapsulation with tannins between 2015 and 2025.

In 2015, relatively few patents were granted (3) and applications were filed (7), reflecting the early exploitation of this technology. However, from 2016 to 2020, a substantial increase in patent applications occurred, with notable peaks in 2019 (13 applications) and 2020 (17 applications). This growth may indicate an increasing interest in microencapsulation technology and intensified research in this area. It is important to note, however, that the apparent year-to-year fluctuations are accentuated by the relatively small number of total records analyzed, and should therefore be interpreted as indicative of emerging trends rather than statistically significant variations.

However, granted patents did not keep pace with the applications. Granted patents remained relatively constant, with peaks in 2017. This mismatch may reflect the approval process timeframes and complexity of the submitted patent, often requiring detailed analysis before granting.

In 2021, the number of granted patents decreased to 1, whereas applications remained significant (11). This decline may be attributed to bureaucratic obstacles or technological adaptations that require time to be recognized as patentable.

In more recent years (2022–2024), patent applications increased, with 2023 notable for 20 applications. Although granted patents have not matched this increase, the greater application numbers suggest substantial innovation potential, reflecting continued interest and pursuit of intellectual protection in this field.

For 2025, with one patent application in progress, further advances may emerge, potentially indicating accelerated development and application of microencapsulation technology in the years to come.

Analysis of keywords in published articles (Figure 3B) reveals the primary focus areas of scientific studies. Among the most recurrent terms are “Agronomy,” “Biochemistry,” “Biology,” “Food Sciences,” and “Animal Sciences,” with “Rumen” also prominently featured. This reflects the multidisciplinary nature of the technology but also suggests a degree of fragmentation in its application and investigation. There remains a need to align biological efficacy, particularly in ruminal systems, with technological development and patent strategies. Many studies emphasize physicochemical or environmental dimensions, yet biological validation under field conditions and the long-term impacts on animal performance appear to be underrepresented.

While patent filings are numerous, scientific publications have not kept pace. This discrepancy may reflect a limitation in knowledge dissemination, as patent databases are less integrated into traditional academic search platforms and often use technical–legal language that hinders interpretation. Consequently, patents are less frequently examined by researchers, reducing their potential to inform or inspire further scientific investigations and publications. Nevertheless, it presents opportunities to investigate unresolved issues in ruminant supplementation with microencapsulated lipids and tannins. Further exploration of different tannin sources, structural classifications (condensed or hydrolyzable), and their synergistic interactions with lipid matrices remain limited. These factors are critical to understanding release kinetics, bioavailability, and potential additive or antagonistic effects *in vivo* (70). Therefore, transitioning from innovation registration to applied scientific validation may represent a valuable step toward advancing scientifically grounded innovations that are scalable, economically viable, and regionally adaptable within ruminant nutrition.

Although the technological benefits of lipid and tannin microencapsulation—such as improved nutrient protection, controlled release, and oxidative stability, are well established, few studies have assessed their economic feasibility at commercial scale. Analyses in related feed systems, such as rumen-protected amino acids, indicate that higher production costs can be partially offset by improvements in nutrient utilization and milk yield (71). Similarly, process-level assessments of spray-drying operations estimate encapsulation costs in the range of US $0.20–0.25 per kg, depending on equipment scale and solids content (72). However, recent reviews emphasize that specific techno-economic studies for lipid and tannin microencapsulation in ruminant nutrition remain scarce, highlighting the need for applied validation to balance technological, environmental, and economic sustainability (4, 73).

### 3.6 Gaps and future prospects

Comprehensive evaluations of the anti-nutritional effects of tannins and the physicochemical stability of their complexes are necessary to determine optimal inclusion levels and post-ruminal lipid absorption dynamics (74). These investigations are crucial for refining the utilization of tannins in ruminant diets, particularly when considering individual variability in metabolic responses and feeding behavior (75, 76). Moreover, this perspective reinforces the importance of *in vitro* models that simulate not only internal ruminal conditions but also external environmental variables related to the storage stability of encapsulated compounds, such as temperature, humidity, and oxidative exposure (70), thereby ensuring greater translational accuracy of experimental results. However, many current in vitro systems still fall short in replicating the dynamic nature of the rumen environment, particularly in terms of microbial adaptation, motility, and compound degradation pathways, which are limitations that hinder their predictive reliability for evaluating encapsulated compounds (77).

Although spray drying is widely recognized and used across industries for microencapsulating bioactive compounds, its effectiveness in protecting feed lipids from oxidation and ruminal degradation remains unexplored, mainly in ruminant nutrition. Understanding the ideal microencapsulation conditions, including process parameters, wall material properties, and interactions with dietary components, is important for evaluating the applicability and efficiency of this technology under practical animal feeding conditions. Microencapsulation has been consistently highlighted as a promising approach that creates a physical barrier, protecting lipids against adverse factors such as oxygen exposure and the ruminal environment (12, 27, 47). Additionally, it enhances compound stability and enables targeted release throughout the gastrointestinal tract, as evidenced by several studies (78, 79).

Despite its potential, considerable knowledge gaps persist, particularly regarding the stability of microencapsulated lipids during rumen transit and their actual influence on digestibility and animal performance (10, 80). Further investigation is required to elucidate how the physicochemical characteristics of microencapsulated systems influence the release of kinetics and subsequent metabolic utilization. In this context, studies should also evaluate the efficiency of bioactive compound absorption and their functional outcomes *in vivo*. Additionally, the co-microencapsulation of tannins with other additives such as essential oils, probiotics, or enzymes may offer synergistic benefits by combining functional properties and enhancing ruminal modulation (81).

It is also necessary to explore the long-term effects of microencapsulated lipids and tannins on rumen health and fermentation dynamics, as well as their potential contribution to environmental mitigation strategies, particularly in reducing methane emissions. Research efforts must continue to optimize spray drying protocols, including drying temperature, solid content, and encapsulation efficiency, and assess how such adjustments impact animal productivity and product quality. In parallel, assessments of production costs, microencapsulation yield, and process scalability are required to support the commercial feasibility of integrating these technologies into feed manufacturing systems (56). Moreover, future research should explore how microencapsulation strategies can be tailored to different breeds, physiological stages, and production environments, particularly in pasture-based systems, where precision nutrition remains a significant challenge.

From a sustainability perspective, tannin-based and lipid-based microencapsulation systems represent promising tools for promoting environmentally responsible livestock practices by reducing reliance on synthetic additives and improving nutrient utilization efficiency. However, their efficacy may vary depending on the source, dose, and interaction of the microencapsulated compounds, as well as the physiological status of the animals. The increasing reliance on preliminary studies and *in vitro* evidence underscores the need for more robust *in vivo* trials across diverse production systems and climatic conditions to ensure the reproducibility and scalability of results.

In summary, the continuous development of innovative microencapsulation technologies presents significant potential for addressing modern challenges in ruminant nutrition. By addressing key gaps in stability, bioavailability, environmental performance, and scalability, these strategies can support the design of more resilient and sustainable animal production systems.

To further advance the integration of microencapsulation in ruminant nutrition, future studies should also consider emerging technologies, such as spray-freezing-drying and multilayer coating systems, which have shown promise in enhancing microencapsulation efficiency and controlling the release of bioactive compounds (82). These approaches may offer enhanced thermal protection, improved matrix stability, and better control over release kinetics under gastrointestinal conditions. Advances in nano and microencapsulation spray-drying techniques, including droplet size control and optimization of wall material formulations, have demonstrated the potential to refine microencapsulation strategies in terms of yield and scalability (83). Such technological progress, combined with supportive sustainability-oriented public policies and incentives, can accelerate the large-scale adoption of tailored microencapsulation systems, making them both economically viable and environmentally strategic.

## 4 Conclusion

This review emphasizes the importance of nutritional strategies in ruminant production systems, highlighting the potential of innovative technologies, such as microencapsulation, to improve digestibility, nutrient utilization, and overall feed efficiency. Effective nutritional management contributes not only to improved animal health and productivity but also to the reduction of environmental impacts, particularly through the mitigation of enteric methane emissions. Bibliometric analyses revealed a growing scientific and technological interest in this field, particularly through the increasing number of publications and patent applications related to the microencapsulation of bioactive compounds in ruminant nutrition.

Among the promising strategies, spray-dried microencapsulation of lipids stands out for its capacity to form protective matrices that stabilize these compounds against oxidative degradation and ruminal biohydrogenation. Incorporating natural co-materials such as tannins has shown synergistic effects by enhancing the physical stability of microparticles while contributing to rumen modulation and improved compound bioavailability.

Despite advances, key knowledge gaps persist regarding the in vivo assessment and long-term physiological effects of microencapsulated systems. Standardized evaluation protocols, especially under varying diets and environmental conditions, remain limited, and the economic feasibility of large-scale application is still uncertain. Future research should focus on optimizing spray-drying parameters, characterizing tannin type and concentration effects and their interactions with other formulation components, and validating biological efficacy under field conditions through integrated, multidisciplinary approaches linking food, animal, and environmental sciences.

In conclusion, the advancement of microencapsulation technologies for lipid delivery in ruminant diets represents a promising frontier to meet contemporary challenges in animal nutrition. By addressing current limitations in stability, controlled release, and practical applicability, these strategies can significantly contribute to the development of more efficient, resilient, and environmentally responsible production systems.
